# The Alcohol Question

**Published:** 1862-01

**Authors:** Daniel Hooper


### THE ALCOHOL QUESTION.

By DANIEL HOOPER, B. A. & M. B.

How do alcoholic liquors act on the human body—as foods or as poisons?—and is their regular and moderate use beneficial or injurious? These are questions to which our most eminent physicians and physiologists give contradictory answers, and the intelligent and reading classes appeal to them in justification of their habits of indulgence or abstinence; they take or avoid alcoholic liquors systematically, and on principle, and the practice in either case is based upon a scientific truth or a scientific error. It is therefore of the highest importance, both for the sake of the public health and of science, that those to whom we naturally look for guidance in these matters should come to some definite and determinate conclusions on the alcohol question. What can be more deplorable than the fact that the medical profession is divided into two distinct schools on this subject—the one represented by Dr. Carpenter, the other by Dr. Todd, Dr. Chambers, etc.? By one school, alcoholic drinks are regarded as *food*, by the other as *poison;* by one as a regular, daily, and useful, if not necessary, part of man's sustenance; by the other as powerful medicinal or poisonous agents, only useful, like those of the apothecary, under certain rare and peculiar circumstances. One school would place them in the same category as salt, sugar, butter and tea, as articles of ordinary domestic consumption; the other would range them on shelves, in company with laudanum, salt volatile and chloroform. These facts prove that a deeply-rooted fundamental creed as to the action of alcohol upon the human tissues lies at the bottom of, and gives its peculiar tinge or complexion to, each of these schools; and until this action of alcohol upon the tissues and vital functions is finally settled and agreed upon, it is impossible that the teaching of men of science and the practice of the public can be, as in the case of meat and bread, unanimous and satisfactory.

It is extremely difficult to determine, with accuracy and precision, the mode in which any agent affects the bodily tissues and functions, but experience and experiment, induction and deduction, chemistry and physiology, enable us, in many cases, to arrive at the truth, or an approximation to it; and, consequently, there is at the present day a tolerable, constant and unanimous belief respecting the *modus operandi* of a considerable number of dietetical and medicinal agents, and it is much to be lamented that two totally opposite and contradictory beliefs should exist on a subject of such vital importance to the public as that of the action of alcoholic liquors upon the human body. I do not pretend to be able to settle this great and difficult question, but I shall endeavor, by quotations from high authorities, as well as by observations and reasonings of my own, to exhibit fully and clearly its present state, and thus to render its solution easier and less distant.

Alcohol appears to seek out and fix upon *nervous* matter, and to act directly and specially upon it, just as other agents localize themselves in particular organs. Dr. Todd regards alcohol as a *food*—Dr. Carpenter as a *poison*—to nervous matter; both, however, agree that it acts upon the nerve-cell and fibre *directly*, and upon the encephalon almost to the exclusion of the spinal cord. "So far as it influences the nervous system," says Dr. Todd, "the action of alcohol is that of a stimulant—an unfortunate term, indicating a distinction without a difference; other forms of food are likewise stimulant, but as they do not act directly and quickly upon the nervous system, their exciting properties are not so apparent. In like manner, alcohol possesses its stimulating property, because it is a form of aliment appropriated to the direct nourishment of the nervous system, and to its preservation; its special adaptation to this system gives it an immediate exciting power superior to any other kind of food." He further says, that alcohol, even when taken in excess, does not produce inflammation of any organ, but that its bad effects are shown in the nervous system; it damages the nutrition of the nervous matter, poisons the nerve-fibre and nerve-cell, and produces anæmia of the brain. Waste of nervous matter is indicated by tremblings and impaired mental power, and these symptoms may be caused by mental anxiety, fatigue, or sexual excesses, with or without the use of alcohol. Todd contends that the proper and moderate use of alcohol repairs and envigorates the nervous system; in short, he regards it as "*its appropriate pabulum*," or food; if given beyond what is required in the treatment of disease, he says it will be exhaled and perceptible in the breath; not so, if the quantity be proportioned to the wants of the system. Dr. Marcet, in a book entitled "Chronic Alcoholism," has very well described the effects of the habitual and excessive use of alcohol. The symptoms, he says, may appear during the indulgence, or long after the discontinuance of the bad habit, and are these: Headache, vertigo, unsteady gait, tremors, musæ volitantes, ringing in the ears, deficiency of what the French call "aplomb," nervousness, sleeplessness, night-mares, frightful dreams, weakness in the loins, hips and knees, inability for exercise, and weakness of intellect and memory. The same or similar symptoms may arise from other causes besides alcohol; such as mental anxiety, excessive intellectual toil, sexual excesses, inordinate smoking, etc., all which, in common with alcoholic excesses, exhaust or waste the nervous matter. Dr. Todd, as I said before, regards alcohol as the proper pabulum of the nervous, in the same sense as albumen is the appropriate pabulum of the muscular tissue. Carpenter, on the other hand, endeavors to show that alcohol is essentially destructive, of nervous matter; he contends that it only stimulates the brain and nerve as the spur does the horse, and that this stimulation wastes and destroys it, so that in the end, and on the whole, it will be in worse condition than before, and will require time and repose in order that it may be fed and repaired by blood containing what he considers to be its appropriate pabulum—viz: phosphorized fats, albumen, etc. If every act of the mind, every thought and emotion, wears away some portion of the nervous matter, just as every muscular contraction wears a part of muscular fibre, it is evident this must, in both cases, be restored in some way. Now Carpenter contends that alcohol cannot, and does not, restore nervous matter, but that it only stimulates the wasted and jaded brain and nerves to further efforts—that is, in fact, it acts upon them as the whip or spur does upon the jaded horse, making them work at the expense of still further wear and tear; so that alcohol, on this view, is a sort of suicidal instrument to the nervous system, goading it on to its own destruction. What then, can and does repair nervous waste? Dr. Todd would answer, "Above all things, *alcohol*." Dr. Carpenter would reply, "Certainly *not alcohol*, but rest, sleep, and a blood containing the proper pabulum of nervous matter—fats, albumen, etc. No doubt there are many agents which all the world admits, *do* repair nervous waste; such are tea, coffee, fresh air, recreation, sleep, and good food, about which there exists no sort of doubt or question; but good reasons ought to be given for excluding alcohol from the category. Now, of tea, coffee, exercise, study, sleep, etc., we may affirm, that used within certain limits, they stimulate, strengthen, nourish, and repair the nervous tissues; and that beyond these limits, they weaken, depress, and waste it. May this not be asserted, also, of alcohol? Dr. Carpenter argues that alcohol cannot ultimately benefit nervous matter, because it is incapable of regenerating it—i. e., of becoming its material pabulum, or food; but no one questions the benefit of tea, coffee, moderate study, sleep, and recreations; and yet we have no reason to suppose that they, *per se* and directly, contribute the most minute particle of matter to the brain and nerves.

In studying the physiological action of alcohol upon the human body, we must never forget that it is one of that large class of agents whose influence varies, not simply in amount, but kind or quality, according to the quantity administered; so that the effects of a large dose will be, not a mere *multiple* of those of a small one, but of a totally different character. In some few cases, as those of lying or stealing for instance, quantitative difference does not produce qualitative difference; but in the majority of cases it does. A certain temperature produces ice—a higher one steam; a certain weight bends a spring—a heavier one weakens it; a few hours' study may innervate the brain—a few hours more will enervate it. And may not, also, a certain amount of alcohol, tea, coffee, etc., strengthen the nervous system, and a larger one weaken it? Or is alcohol mischievous in all proportions, whilst tea, coffee, study, etc., are not so? Cause must be shown why alcohol is to be excluded from the class of agents which do good in moderation, and harm in excess.

Is alcohol a *food* or a *poison?* This question is still an open one; the end and aim of all food is force; food is finally converted into force, which may be regarded as its true definition, and almost as its equivalent, convertible, and equipollent term. In this sense, alcohol, tea, study, exercise, oxygen, may all be regarded as food, for they all give force although they probably do not, directly, and *per se*, furnish any material pabulum to the brain and nerves. Alcohol, however, may possibly do so, (as we shall see in another place,) and may, therefore, be regarded as a more real food than the others. Moderate exercise of mind and body is a generator of force, and the indirect means of imparting growth and strength to the brain and muscles. But alcohol is more really and strictly a food than any such agent as exercise; for all material food is either plastic (tissue-making) or respiratory (heat-making,) and alcohol is a most excellent respiratory or calorific food, for it is far more digestible, and far quicker in its action than starch, fats, and sugar, and is at once absorbed by the vessels on the walls of the stomach; consequently, where time is an object, as in cases of fainting, or of collapse from accidents, alcohol possesses a manifest advantage over the more solid and slowly-acting hydro-carbons. Even Carpenter admits the rapidity of its action; but he objects to its employment except in cases of emergency. He says, "Alcohol is the quickest in its action of all the hydro-carbons, but others would be equally and more permanently efficacious, if time were only given them to act. In some exceptional cases this *time* cannot be given, and then alcohol is indicated." He also says, "Alcohol, by presenting itself first for combustion in the lungs, prevents the other carbonaceous matter of the blood (supplied from food and other sources) from being burnt off in the lungs; these, consequently, are thrown upon the skin, liver, and kidneys, which organs are very likely to suffer in the performance of this extra duty." All writers agree with Moleschott, that "wine saves the tissues from being burnt, by offering itself as a fuel;" and the most recent experiments of the eminent physiological chemists of Germany have completely established this truth; that alcohol, (in common with tea and some other agents,) by preventing the waste of the tissues is, if not a real and material pabulum, at least an equivalent to it, a diminution of expenditure being, of course, tantamount to an increase of income. It is objected, that alcohol is only a temporary stimulus; that the force generated by it is only temporary. But this is not a valid objection, since all stimulus, all force is temporary; food, fresh air, exercise, are all stimuli, or generators of force, but are temporary in their action. Life is only possible under incessant stimulus. Tea and coffee are called "agreeable and refreshing" stimuli; why should the stimulus of alcohol be called noxious? What is there peculiar in the alcoholic stimulus that demarcates it from all others? If a moderate quantity of tea be taken, the effect is agreeable and refreshing; but if taken in excess, the effect is disagreeable and enervating. We may say the same precisely of a mountain walk; neither of these stimuli leaves the body in a worse condition (but, on the contrary, a better) at the expiration of five or six hours, than it would have been without it. Dr. Chambers applies this test (which seems to be a very fair one) to alcohol, and contends that if an individual finds himself better able to perform all the duties of life during five or six hours' interval of his meals with and without alcoholic stimulants, then they are good for him; at the end of this interval, if alcoholic liquors agree with him, he will feel more cheerful and vigorous than he would have felt if he had not taken them.

In the case of poisons, or alcoholic excesses, this average interval of five or six hours would be one of misery, and before its expiration the poison or the stimulus would demand imperatively either a remedy or a repetition.

All physiologists agree, that every mental effort wears away a portion of nervous matter, just as every muscular effort destroys and removes the muscular fibre. This being so, the lost matter must, in both cases, be restored by the blood, unless a totally different law obtains in the two structures, which is improbable. Now, we know that the muscular tissue is repaired by the albuminous matter of the food existing in the blood. What part of the food, then, is it which, entering the blood, repairs, restores, or builds up the effete nervous matter? Phosphorous, oils, and fat constitute a large portion of the nervous structures; and alcohol, being a hydro-carbon, is chemically allied to these components of nervecoll and fibre. But what is its precise action upon them? Is it a real pabulum? Does it nourish them, directly and materially, and build them up, in the same sense as albumen does the muscular tissue? Or does it merely affect them after the manner of study, or cheerful amusement, without imparting anything to them of a real and substantial character? Or, lastly, does it simply quicken the circulation, and so send a larger amount of blood to the brain and nerves in a given time? These and similar questions have yet to be answered before the subject of alcohol can be well understood.

My own observation and reflection may lead to believe that alcohol drinks are highly useful, if not necessary, articles of regular, daily consumption, for vast numbers of persons; but that their kind and amount must be determined by age, sex, constitution, mode of life, and other circumstances. I believe they are more necessary for those whose avocations involve head-work, anxiety, and wear and tear of the brain, than for such as lead a comparatively animal life, or one of mere bodily labor. And I think it will be found that the degree of refinement of alcoholic liquor required is in tolerable exact ratio to the expenditure of brain-powder. The agricultural laborer, for example, is satisfied with ginger-beer, or very poor home-brewed beer; clerks and shopkeepers with bitter ale; and barristers, judges, and members of Parliament with wine. In fact, we find a gradation of brain-work corresponding pretty exactly to that of the refinement and alcoholic powder of the liquor habitually and instinctively made use of. On the continent, also, we see illustration of the same fact—the strength and refinement of the wines consumed, gradually rising with the exaltation of the brain-work of the consumers. Nor is it owing, as might be supposed, entirely to difference of rank or pecuniary resources; for every man finds the same fact illustrated and corroborated in his own experience. We all find, when on our tours in Switzerland or the Highlands, where we enjoy pure air, good food, and rest and recreation of brain; when, in short, we are living an animal rather than an intellectual life, we care nothing for, and do not require any sort of, alcoholic liquor; whereas, when engaged in our profession or business in London, in the midst of bad air, noise, hurry, bustle, competition, and excitement, we are conscious of an unmistakable craving for a certain amount of alcohol with our daily food; the reason being that, in the one case, we are doing everything to refresh and fortify, and in the other to exhaust and wear out, the nervous system. This fact goes far to prove that alcohol, in some peculiar but as yet unexplained way, *does* repair nervous tissue.

In estimating the value of alcohol, the experience and testimony of healthy persons who use it habitually, and in moderation, ought to be taken into account; also the fact that all ages, and in every corner of the globe, man has discovered a method of preparing it. There are persons who do very well without alcohol; but this is no proof that it is useless to others. There are country districts where the laborers are healthy and strong without meat, and with beer almost as weak as water; but does it follow that the same fare would suit the London lawyer, barrister, judge, or member of Parliament? No, the two cases are totally different. Men whose labor resembles horses may and do live, like horses, upon corn and water; but those who are calculating, thinking, and reasoning, twelve hours out of twenty-four, require a more refined sort of food and drink. A ploughboy will look fat and rosy upon his bread and cabbage and hard pudding and water; whilst a Gladstone will require, besides these, good animal food, tea, coffee, and an alcoholic liquor of great purity and refinement. If the brain-work of the London clerk demands a supply of Bass's ale, that of the working statesman will require something approaching œnanthic æther!

Two arguments used by total abstainers require a short notice. They maintain that alcoholic liquors cannot afford any real and permanent benefit, because they contain little or nothing of a solid nature (as proved by evaporation to dryness.) But if this proves the worthlessness of wine, so does it of tea and coffee. The fact is, experience has proved that all these agents, in spite of their unsubstantial nature, do refresh the wearied brain and nerves, and impart new life and health and spirits. Exercise, fresh air, study, tea, coffee, and segar smoke, are all devoid of solidity; but the argument that they are therefore incapable of imparting anything to the human body is still more so. On the contrary, we know that exercise does add bulk and weight and substance to the muscles, that fresh air does redden and enrich the blood; that recreation and study do nourish the brain and nerves; that tea, coffee, and alcohol, do, at any rate, prevent waste of the tissues (and probably also directly nourish the nervous system,) and that moderate smoking, by soothing and calming the over-busy and excited brain, prevents its exhaustion and waste; in short, some of the least material agents have the most real, powerful, and beneficial influence upon the human body. Again teetotalers contend that, in the case of alcohol, it is impossible, to define moderation and excess, since what is moderation to one man is excess to another, and vice versa; but this is equally true of salt, tea, sugar, coffee, and many other things, moderation and excess in which they regard as tolerably well defined by common consent. The truth is, there is a certain recognized standard quantity of alcohol, salt, tea, sugar, coffee, etc., which all men agree to call moderate, and the difficulty is not greater in the case of alcohol than of any other article of daily consumption. The man who eats a leg of mutton at a meal, or consumes a pound of salt, or drinks a gallon of beer per diem, is looked upon by the public as a monstrosity, an exception, a wonder, whilst he whose daily consumption is one sixteenth of these several articles is regarded as an ordinary individual—a type of the masses; in short, the excessive and the moderate man are as well known and as easily recognized as are any of the types and their deviations in the animal and vegetable world. It is idle and absurd to pretend that the boundry line between moderation and excess is indefinable. I believe every man knows where it is, and when he has overstepped it, even although, from long habit and sensibility, the transgression may have little effect upon him. The soldier's rations and the diet-lists of great hospital, are so many proofs that there is a standard in these matters, well understood, and that public institutions, in their dietetical arrangements, do not contemplate or provide for monsters who eat a leg of mutton or drink three gallons of beer per diem.—*London Lancet.*

